# Real-world study of efficacy, risk management and reasons for discontinuation of natalizumab for treatment of multiple sclerosis in Russia

**DOI:** 10.1371/journal.pone.0217303

**Published:** 2019-05-28

**Authors:** Evgeniy Evdoshenko, Alexandra Stepanova, Maria Shumilina, Maria Davydovskaya, Natalia Khachanova, Nikolay Neofidov, Ivan Kalinin, Ekaterina Popova, Ekaterina Dubchenko, Natalia Pozhidaeva, Andrey Volkov, Stella Sivertseva, Anna Prilenskaya, Nadezhda Malkova, Denis Korobko, Ilona Vergunova, Sergey Shchur, Gleb Makshakov

**Affiliations:** 1 SPb Center of Multiple Sclerosis and AID (SBIH City Clinical Hospital #31), St. Petersburg, Russia; 2 State Budgetary Institution of Moscow City “Clinical Trials and Healthcare Technology Assessment Scientific-Research Centre of Moscow Department of Healthcare”, Moscow, Russian Federation; 3 Pirogov Russian National Research Medical University, Moscow, Russia; 4 City Clinical Hospital # 24, Moscow, Russia; 5 City Clinical Hospital # 81, Moscow, Russia; 6 City Clinical Hospital # 71, Moscow, Russia; 7 “Medical Sanitary Unit «Neftyanik», Tyumen Multiple Sclerosis Center, Tyumen, Russia; 8 State Novosibirsk Regional Clinical Hospital, Center of MS and other AID of Nervous System, Novosibirsk, Russia; 9 State Novosibirsk Medical University, Novosibirsk, Russia; 10 City Clinical Hospital # 15, Moscow, Russia; University of Ioannina School of Medicine, GREECE

## Abstract

**Background:**

NTZ is approved in Russia for the treatment of highly active relapsing remitting multiple sclerosis and is reimbursed via federal budget program. However, no data about NTZ treatment in Russia and the effect of federal reimbursement have been performed so far.

**Objective:**

To characterize the population of patients receiving natalizumab and assess the efficacy and risk-management plan (RMP) implementation of NTZ therapy in routine clinical practice in Russia.

**Methods:**

We analyzed data for 334 patients, who received at least one infusion of NTZ. Relapse rate, MRI activity, NEDA-3 status after 2 years were assessed. Anti-JC virus antibodies status and RMP implementation were evaluated. Drop-out rate and reasons for therapy discontinuation were analyzed.

**Results:**

Patients switched to natalizumab in Russia are mainly female (63%), with median EDSS score of 3.5 and high disease activity: 93% had at least 1 relapse and 58% had both T1Gd+ and new T2 lesion a year before therapy initiation. Introduction of federal reimbursement allowed patients with less relapses to start therapy with natalizumab. The only predictor of 6-month progression was EDSS score at the baseline of therapy (HR = 2.1375, 95%CI 1.0026–4.5570, p = 0.0492). 82% patients reached NEDA-3 at 24 month of therapy. 25% of patients discontinued NTZ for reasons: tolerability (14.5%), JCV antibody status (61%), and patient’s decision (17%). RMP was implemented in only 36% patients.

**Conclusion:**

Natalizumab appeared to have high efficacy in Russian clinical practice. Federal reimbursement allowed less active patients to start natalizumab. More efforts should be done to improve RMP implementation.

## Introduction

Multiple sclerosis is a major medical and social problem in Russia with up to 150 000 people affected [1]. Administration of highly efficacious disease modifying drugs (DMDs), such as Natalizumab (NTZ) can significantly improve disease outcomes and patients’ quality of life [2].

NTZ was approved in the Russian Federation in 2010. In 2011, the risk management plan (RMP) for NTZ in Russia was established [3]. In 2012–2013, a prospective, open, non-randomized study on safety and efficacy of NTZ in Russian population of patients with relapsing-remitting multiple sclerosis was conducted (RUS-TYS-11-10158, Clinicaltrials.gov: NCT02142205) [4]. This study has shown efficacy of NTZ comparable with phase III studies and a relatively good safety profile [4]. Recommendations for therapy escalation and updated RMP were published in 2016 and allowed to stratify patients and determine those with high disease activity for further therapy with NTZ [5].

However, due to specificity of therapy reimbursement, NTZ has been hardly accessible for the majority of patients until 2016. In 2016, NTZ has been introduced into the Federal Budget Program ‘7 high- cost nosologies’ and became accessible to a wider range of patients.

Even though NTZ has been registered in Russia in 2010, no multi-center observational studies investigating the effect of NTZ in a routine clinical practice have been conducted. The real profile of patients, receiving NTZ in Russia is currently unknown. Also, the impact of NTZ availability due to pharmaceutical benefits has not been studied. An audit study of RMP compliance would also be highly important for healthcare regulators, however, to our knowledge, no such studies were done.

The aim of this study was to assess the efficacy and certain issues of safety of NTZ therapy in a routine clinical practice in Russia. Study objectives were: 1) to describe the profile of patients that receive NTZ in Russia in routine clinical practice; 2) to assess the efficacy profile of NTZ regarding relapse rate, disability progression and MRI activity during 2 years of therapy; 3) to assess the difference in subpopulations of patients that were escalated on NTZ before 2016 and after 2016 under the auspices of the Federal Program; 4) to assess reasons for and consequences of NTZ therapy discontinuation; 5) to evaluate the RMP compliance.

## Materials and methods

Data were collected in 2018 from 7 multiple sclerosis centers in 4 regions: Saint-Petersburg (Center of Multiple Sclerosis and AID at SBIH City Clinical Hospital #31); Moscow (City Clinical Hospital # 81, City Clinical Hospital # 71, City Clinical Hospital # 15, City Clinical Hospital # 24); Tyumen (Multiple Sclerosis Center in Medical sanitary unit «Neftyanik»); Novosibirsk (State Novosibirsk Regional Clinical Hospital, Center of MS and other AID of nervous system).

The study was approved by the Ethics Committee of the State Budgetary Institution of Healthcare Saint-Petersburg City Clinical Hospital #31. All patients provided their written informed consents.

Patients that received at least 1 infusion of NTZ were included into the study. No exclusion criteria were used.

This was an observational cross-sectional study with retrospective data analysis. The number of patients included was characterized according to the percentage of total patient population receiving NTZ in Russia, which was assessed by total amount of purchased NTZ in 2018 according to the official resources [6].

All study sites were requested to fill in the data matrix. The matrix included issues about patients’ disease characteristics and NTZ therapy and was filled in with the information from the local databases and registries. We collected demographical data, duration of the disease, type of therapy and its duration prior to NTZ. Duration of NTZ therapy, cases of therapy interruption or permanent discontinuation were also evaluated.

Efficacy analysis was performed using the information about the number of relapses during 24 months prior to NTZ initiation and every 12 months while on NTZ. Relapses prior to NTZ were divided into -24 to -12 months and -12 to 0 months. The definition of relapse was unified across all centers. A defined relapse was registered and included into the analysis in case of acute or subacute appearance of new or worsening neurological symptoms lasting longer than 24 hours without signs of fever. Brain MRI scans were performed using standard protocols (T2, FLAIR and T1 post-gadolinium weighted images) on 1.5 T and 3.0 T machines, based on international recommendations [7]. The slice thickness was comparable between consecutive scans and was defined as ≤3 mm [7]. Consecutive scans from 1.5 T and 3 T machines were analyzed separately to avoid misinterpretation. The comparative analysis of data received from 1.5 T and 3 T was not performed since this was not the primary goal of the study. Enlarging lesions were considered as new. MRI data (number of new T2 and T1 Gd+ lesions) was analyzed for 12 months before NTZ initiation and every 6 months while on therapy. Since the interval between regular neurological assessments may have been different between sites, we analyzed the EDSS score every 6 months. 6-months confirmed disability progression (CDP) was assessed using a 6-months roving reference score described by Kappos et.al. [8]. Disability progression was confirmed in case of EDSS score increase on ≥1.0 point for patients with baseline EDSS ≤ 5.0 and in case of ≥ 0.5 point increase for patients with baseline EDSS >5.0.

NEDA-3 was assessed at year 2 in cases where all data regarding the number of relapses, EDSS progression and new lesions on MRI were available.

Safety analysis was performed with regards to the RMP compliance, reasons for NTZ discontinuation and anti-JCV antibody index. For that, we analyzed the proportion of patients for whom the RMP was fully implemented at year 1 and 2 according to Russian recommendations [5]. Information about anti-JCV antibody index was inquired for every 6 months and recorded if available.

Statistical analysis was performed using Statistica 13.0 (Statsoft) and GraphPad Prizm 7.0 (GraphPad Software, Inc). Komogorov-Smirnov test was used for the assessment of normality. Data are presented as median [LQ-UQ; IQR] is case of non-normal distribution and as mean (±SD) for normally distributed data. Mann-Whitney test was used for comparison of non-normally distributed data sets. Paired t-test and Wilcoxon matched-pairs signed ranked test were used for subgroup analysis depending on the type of distribution. Pair-wise non-parametric ANOVA was used for efficacy analysis. Cox proportional regression model, including age, sex, EDSS score at baseline, disease duration and number of relapses during 2 years prior to NTZ initiation was used for the analysis of factors, contributing to the 6-months CDP.

## Results

### Patients’ baseline characteristics prior to NTZ therapy

In total, 334 patients that received at least 1 infusion of NTZ, were included into the study. According to the official resources of the Russian Ministry of Health, this sample size presents 30% of the total Russian population of patients on NTZ [6]. Demographic data are presented in the Table 1.

**Table 1:** Demographic and disease characteristics of the study population.

**Total study participants, n**	**334**
**Male to female ratio**	1: 1.74
**Age at the time of inclusion, years, mean ±SD**	32.94 (± 8.80)
**Age at disease onset, years, mean ±SD**	25.25 (± 8.40)
**Disease duration, years, median [LQ, UQ, IQR]**	6.30 [3.53–10.59; 7.06]
**EDSS at NTZ onset, score, median [LQ, UQ, IQR]**	3.5 [2.5–4.5; 2.0]

EDSS—Expanded Disability Status Scale; NTZ—natalizumab;

#### Disease activity

12 months prior to NTZ, 24/334 (7.19%) patients had no relapses, 87/334 (26.05%) patients had 1 relapse, 101/334 (30.24%) patients had 2 relapses and 122/334 (36.52%) had 3 or more relapses. The median number of relapses 12 months prior to NTZ was 2.0 [1.0–3.0; 2.0], and the same was for the period of 12 to 24 months before NTZ—2.0 [1.0–3.0; 2.0]. For 217 patients MRI was done during 12 months prior to NTZ: 125/217 (57.6%) had at least one new T2 brain lesion, 177/217 (81.57%) had one or more T1 Gd+ lesions. In total, the number of patients demonstrating active disease at baseline with both relapses and new T2 and/or T1 Gd+ brain lesions on MRI was 169/272 (62.13%).

### Prior treatment

**Fig 1:**
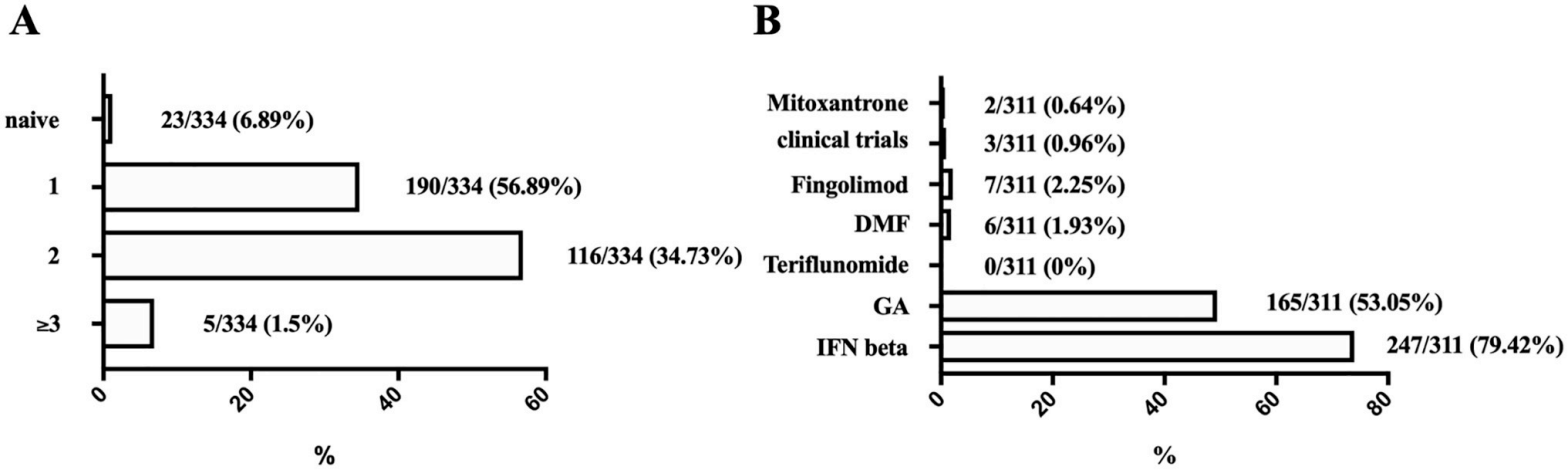
Characteristics of the previous therapy. A—number of previous DMTs; B—profile of previous DMT before NTZ initiation (n = 311, excluding treatment-naïve patients). DMF—dimethyl fumarate; GA—glatiramer acetate; IFN beta—interferon beta.

311/334 patients (93.11%) were previously treated with one or more DMTs and/or immunosuppressant (IS), while 23/334 (6.89%) patients were treatment-naïve. The median duration of disease-modifying treatment was 33.00 months [15–60; 45]. 39/311 (12.54%) patients received DMT for less than a year, 75/311 (24.12%) patients—from 1 to 2 years, 44/334 (14.15%) patients—for 2 to 3 years, 35/311 (11.25%)—from 3 to 4 years, for 118/311 (37.94%) patients the length of therapy exceeded 4 years. The spectrum of therapies received are presented on the Fig 1.

### Natalizumab treatment data

The median duration of NTZ therapy (recorded for 332 patients, who had certain data) was 17.10 [5.00-24.23; 19.23] months. 2 patients didn’t have certain information regarding the end date of therapy due to participation in the RUSTYS clinical trial. 140/332 (42.17%) patients received natalizumab for less than 12 months (20 patients had only one infusion of the drug), 101/332 (30.42%) patients—for 12 to 23 months, 66/332 (19.88%) patients—for 24 to 35 months, and 25/332 (7.53%)—for more than 36 months. The maximal duration of therapy was 79.03 months. In total, all patients received 4611 infusions.

### Subgroup analysis

In our study, we compared two subgroups of patients depending on the time of the therapy switch: before June 2016 (subgroup 1–126 patients) and since June 2016 (subgroup 2–208 patients). The comparison revealed a significant difference in relapse rate: the number of relapses during 12 months and 24–12 months before NTZ initiation was significantly higher in patients from the subgroup 1 (12–0: p = 0.00008; 24–12: p = 0.000001). No other differences were shown for other parameters. The data are shown in Table 2 (below).

**Table 2:** Subgroup comparison data.

Feature	Subgroup 1 (before JUN 2016), n = 126	Subgroup 2 (from JUN 2016), n = 208	p-values
**Sex**	F: 73/126 (57.94%)	F: 139/208 (66.82%)	(Chi-square) p = 0.18
**Age at time of NTZ initiation, years**	31.41 [25.12–38.43; 13.31]	32.02 [27.21–40.12; 12.91]	p = 0.17
**EDSS score**	3,5 [2.5–4.5; 2.0]	3,5 [2.5–4.5; 2.0]	p = 0.65
**Relapse rate in a year prior to NTZ switch**	2.0 [2.0–3.0: 1.0]	2.0 [1.0–3.0; 2.0]	p = 0.00008
**Relapse rate in a 24–12 month prior to NTZ switch**	2.0 [1.0–3.0; 2.0]	1.0 [1.0–2.0; 1.0]	p = 0.000001
**Disease duration before NTZ, years**	6.22 [2.89–10.54; 7.65]	6.32 [3.65–10.39; 6.74]	p = 0.5
**Duration of previous DMT, months**	38 [20–62; 42.]	29 [13–60; 47]	p = 0.15

NTZ—natalizumab, EDSS—Kurtzke’s Expanded Disability Scale Score; DMT—disease modifying therapy. Data are presented as median [LQ-UQ; IQR], if not in % of the whole.

### Efficacy analysis

Relapse rate on NTZ therapy was analyzed for 192/334 (57.49%) patients after 12 months and for 77/334 (23.05%) patients that received NTZ for at least 24 months and had available data. MRI dynamic data was available for 79/192 (41.15%) patients after 12 months and for 25/77 (32.47%) patients after 24 months that received NTZ and had available data. Therapy with NTZ led to a significant decrease of inflammatory activity, based on both relapse rate and MRI-activity. All efficacy data are presented in details in the Table 3.

**Table 3:** Efficacy analysis.

Efficacy parameter	12 months before NTZ	12 months on NTZ	p-value	24 months on NTZ	p-value
**Clinical parameters**					
**Number of subjects analyzed, n**	334	192		77	
**Annual relapse rate, n**	2 [2–2; 0]	0 [0–0; 0]	<0.0001	0 [0–0; 0]	<0.0001
**EDSS score**	3.5 [2,5–4.5; 2.0]	3.5 [2.5–4.5; 2.0]	0.56	3.25 [2.5–4.5; 2.0]	0.08
**MRI analysis**					
**Number of subjects analyzed**	217	79		25	
**Percentage of patients with new T2 lesions**	125/217 (57.6%)	12/79 (15.19%)	<0,0001	2/25 (8.0%)	<0,0001
**New T2 lesions**	1 [0–3; 3]	[0–0; 0]	<0,0001	[0–0; 0]	0.0001
**Percentage of patients with Gd+ lesions**	177/217 (81.57%)	7/79 (8.86%)	<0,0001	2/25 (8.0%)	<0,0001
**Gd+ lesions**	1 [0–3; 3]	0 [0–0; 0]	<0,0001	0 [0–0; 0]	0.0001

NTZ—natalizumab; MRI—magnetic resonance imaging; EDSS—Expanded Disability Status Scale; Gd+—gadolinium enhancing lesions;.Data are presented as median [LQ-UQ; IQR], if not in % of the whole. Results of analysis of 1.5 and 3.0 Tesla MRI are presented as combined.

EDSS progression was assessed at 24 months. For the analysis of disability progression, we included 98 patients that received NTZ for 24 months and those that didn’t but reached the disease progression earlier than 24 months. At 24 months, 8/98 (8.16%) patients reached 6-months confirmed disability progression: 2/98 (2.04%) during first 12 months and 6/98 (6.12%) during 12–24 months after switch to NTZ.

NEDA status was assessed for patients receiving NTZ for not less than 2 years. Analysis was done for 22/77 (28.57%) patients with all data available at the 24 months. NEDA-3 was reached by 18/22 (81.82%) of patients.

### Cox regression analysis of CDP factors

We investigated factors that led to 6-months CDP during NTZ treatment in 95 patients. 8 patients reached the milestone, while other 87 patients were censored. Multivariable Cox regression analysis showed the EDSS score at baseline to be a predictor of 6-months CDP (hazard ratio (HR) = 2.14, 95% confidence interval (CI) 1.0026–4.5570, p = 0.0492). Other factors didn’t show association with disease progression.

### Therapy discontinuation

**Fig 2:**
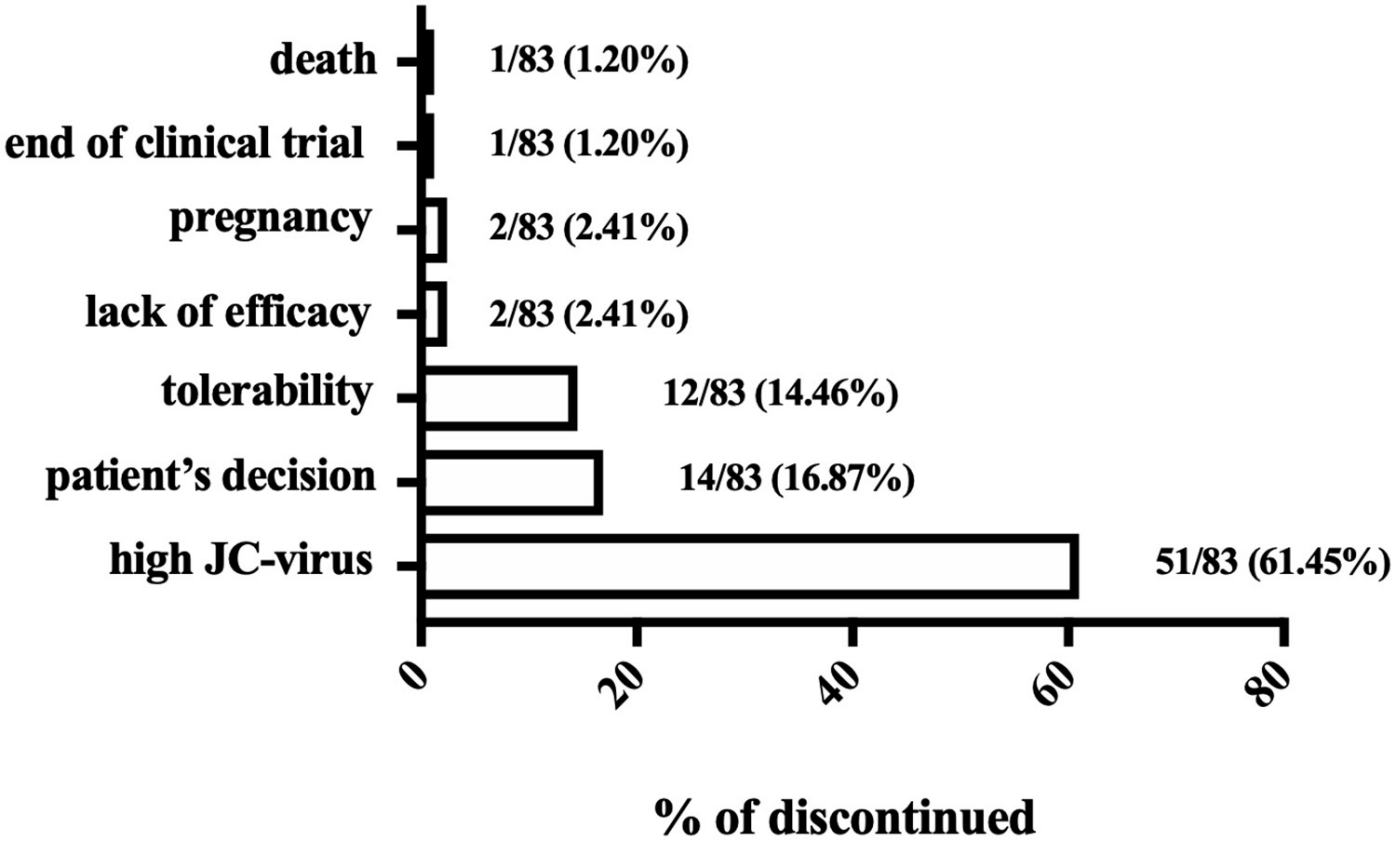
Reasons for natalizumab discontinuation. JC-virus—John-Cunningham virus.

83/334 (24.85%) patients discontinued NTZ. Of them, 5/83 (6.02%) resumed the NTZ later, while other permanently discontinued NTZ. 11/83 (13.25%) patients received only one infusion of NTZ and 16/83 (19.28%) patients stopped therapy during the first year. The mean time of follow-up after stopping therapy was 45.04±20.47 months. During this period 26/83 (31.33%) patients developed relapses. The mean time until first relapse after therapy discontinuation was 7.37±11.16 months. The reasons for stopping therapy are presented in the Fig 2.

### JCV antibody dynamics

305/334 (91.32%) patients had available data at baseline. 90/305 (29.51%) patients were seronegative (JCV-), 41/305 (13.44%) had intermediate titer, 77/305 (25.25%) were seropositive (JCV+), with low titer (index <1.5) and 97/305 (31.8%)–positive with high titer (index>1.5). After 24 months 7/90 (7.78%) JCV- patients had seroconversion.

### Risk management plan evaluation

215/334 (64.37%) patients, who received natalizumab for at least 6 months and had available data, were included into analysis of risk management plan (RMP) compliance. We firstly analyzed the compliance regarding JCV-testing frequency, then we analyzed MRI performance. The risk management plan was fully implemented for 77/215 (35.81%) patients during the whole period of NTZ therapy. Mostly, such a low number of patients was explained by a failure to perform timely anti-JC-virus antibodies testing.

## Discussion

The patients’ profile, efficacy and safety of the drug in routine clinical practice may significantly differ from clinical trials. Also, local healthcare features may predetermine a specific patient’s profile receiving the drug. The profile of patients who were transferred to NTZ in Russia, appeared different from that seen in observational studies, like STRATA, where patients had higher age and disease duration, but lower EDSS score, clinical and radiological activity of MS [9]. However, these results were nearly comparable with RUS-TYS data [3]. These data indicate, that in Russia, NTZ is administered to patients with more aggressive MS and this pattern has not much changed since 2012 [3].

The analysis of prior treatment revealed that a relatively low amount of patients receives NTZ as a first-line therapy and nearly half of patients receive NTZ after 2 or 3 DMTs. This may generally be due to insufficient availability of the drug. Compared to the data of the RUS-TYS trial, the amount of treatment-naïve patients was lower (5.8% in our study vs 16% in RUS-TYS). These data show that the majority of highly active patients are initially treated with standard first-line DMTs [3].

Major disease characteristics didn’t change significantly with improved access of NTZ after introduction of the drug into the Federal Budget Program ‘7 high- cost nosologies’. However, patients included after 2016 had significantly lower number of relapses before therapy escalation. This could, probably, mean that under the federal reimbursement strategy patients can get better access to the drug starting therapy earlier with lower level of MS activity. This may significantly improve their quality of life and diseases outcomes.

NTZ had a great impact on MS activity. Patients on NTZ had a significant decrease of relapse rate after 2 years of therapy. The number of relapses during first and second years of therapy tended to 0, that corresponds with data from other studies [10, 11]. Similarly, MRI activity was almost completely suppressed, proving a strong efficacy of NTZ in highly-active patients [10, 11]. EDSS score was mostly stable during 2 years of therapy, with only 8 patients having 6m-CDP. This data are in line with other observational programs [12]. NEDA-3 was reached by the majority of patients (82%).

High EDSS score at the time of NTZ initiation was shown to be the only factor, predicting 6-months CDP after 2 years. Our data confirm previous findings that patients with low EDSS on NTZ have lower relapses, and, hence, have lower chance for disability progression [12]. NTZ should be initiated as early, as possible in patients with active disease to prevent disability progression.

In this study, the main reason for therapy discontinuations was the increasing risk of PML due to JCV antibody status. Patients’ JCV serology status was relatively stable with seroconversion rate not exceeding 7.78%, comparable with data from other studies [13].

Unfortunately, the RMP compliance was only 36%. This study was not powered enough to study the reasons for such a poor implementation. The process of NTZ therapy needs a better reimbursement strategy and a stronger attention of all participants of the process (neurologists, healthcare regulators, manufacturers) to provide patients with better disease control and safety follow-up. More resources are required to provide patients with timely MRI to implement the RMP. This may decrease the number of patients stopping therapy due to the fear of PML, since patients may be able to continue the drug with more often MRI monitoring. One of solutions in this sphere may be the introduction of electronic medical record systems, timely providing the neurologist with all data and helping in decision-making process. Initiation of therapy for patients with high JCV antibodies index should be discussed and probably avoided. Discontinuation of therapy may lead to disease reactivation and subsequent relapses. Other strategies exist for patients with high anti-JCV antibody index, such as ocrelizumab or alemtuzumab.

The study had several limitations that stem from the observational design of the study. The main limitation was the amount of collected information and missed data. For that reason, the efficacy outcomes were available only for a limited number of patients. Some patients for financial reasons may await MRI for a long period of time (more than 12 months). Since we used limited timelines of 12 and 24 months, these patients may have exceeded these timelines for MRI later and were not included into the analysis. This limitation is related to reimbursement strategies for MRI in Russia. Under routine practice patients performed MRI on 1.5 and 3.0 Tesla. 3 Tesla may detect more lesions due to improved signal-to-noise ratio. In this study, we analyzed only patients, that performed MRI always on one type of the machine consistently across the study. Another limitation of the study was the method for collecting the information. Investigators provided data into a pre-specified matrix and the data were not extracted from the cloud electronic medical records. This may led to some data missed without a possibility for the double check of data consistency. Some queries were generated to researchers by the central analysis team to improve the quality of data.

Natalizumab is a highly efficacious DMT for active multiple sclerosis. Taking into consideration complex issues of therapy management, NTZ should be administered only in specialized MS centers where the physicians have enough resources, experience and knowledge.
